# Enteral siRNA delivery technique for therapeutic gene silencing in the liver via the lymphatic route

**DOI:** 10.1038/srep17035

**Published:** 2015-11-23

**Authors:** Masahiro Murakami, Kazutaka Nishina, Chie Watanabe, Kie Yoshida-Tanaka, Wenying Piao, Hiroya Kuwahara, Yuji Horikiri, Kanjiro Miyata, Nobuhiro Nishiyama, Kazunori Kataoka, Masayuki Yoshida, Hidehiro Mizusawa, Takanori Yokota

**Affiliations:** 1 Laboratory of Pharmaceutics, Faculty of Pharmacy, Osaka Ohtani University, 3-11-1 Nisikiori-Kita, Tondabayashi-shi, Osaka 584-8540, Japan; 2 Section of Molecular Technology, Core Research for Evolutional Science and Technology (CREST), Japan Science and Technology Agency (JST), 4-1-8 Honcho, Kawaguchi-shi, Saitama 332-0012, Japan; 3 Department of Neurology and Neurological Science, Graduate School of Medical and Dental Sciences, Tokyo Medical and Dental University, 1-5-45 Yushima, Bunkyo-ku, Tokyo 113-8519, Japan; 4 Division of Clinical Biotechnology, Center for Disease Biology and Integrative Medicine, Graduate School of Medicine, The University of Tokyo, 7-3-1 Hongo, Bunkyo-ku, Tokyo 113-8656, Japan; 5 Polymer Chemistry Division, Chemical Resources Laboratory, Tokyo Institute of Technology, 4259 Nagatsuta, Midori-ku, Yokohama-shi, Kanagawa 226-8503, Japan; 6 Department of Materials Engineering, Graduate School of Engineering, The University of Tokyo, Bunkyo-ku, Tokyo 113-8656, Japan; 7 Department of Life Science and Medical Ethics, Graduate School of Medical and Dental Sciences, Tokyo Medical and Dental University, 1-5-45 Yushima, Bunkyo-ku, Tokyo 113-8519, Japan; 8 Center for Brain Integration Research, Tokyo Medical and Dental University, 1-5-45 Yushima, Bunkyo-ku, Tokyo 113-8519, Japan; 9 National Center Hospital, National Center of Neurology and Psychiatry, 4-1-1 Ogawahigashi-machi, Kodaira-shi, Tokyo 187-8551, Japan

## Abstract

An efficient targeting delivery technology is needed for functional oligonucleotides to exert their potential effect on the target gene without an adverse effect *in vivo*. Development of enteral delivery systems for nucleic acids is a major challenge because of their large molecular size and instability. Here, we describe a new enteral delivery technique that enables small interfering RNA (siRNA) selectively delivered to the liver to silence its target *Apolipoprotein B* gene expression. A nuclease-resistant synthetic siRNA was conjugated with α-tochopherol and administered as lipid nanoparticle to the large intestine of the mice in a postprandial state. The selective transport into the liver, effective gene silence, and consequently significant reduction in serum low density lipoprotein-cholesterol level, were demonstrated. The chylomicron-mediated pathway via the lymphatic route was suggested as major mechanism. This unique approach may provide a basis for developing oral and rectal delivery systems for nucleic acids targeting liver.

Functional nucleic acids based on RNA interference (RNAi) technology, i.e., siRNAs, are powerful tools for sequence-specific silencing of disease-related gene expression and may have important clinical applications in diseases in which existing therapies are still insufficient^1^,^2^. Many nonviral carriers or vectors have been reported for delivery of nucleic acid molecules, and functionalized polymeric nanospheres and lipid nanoparticles (LNPs), such as liposomes, may be the most promising candidates^3^. Despite some early success in local injections, however, the clinical development of nucleic acids as systemic therapeutics has been stalled due to the lack of a safe and effective *in vivo* delivery technique^2^. We hypothesized that the best *in vivo* carrier for siRNA would be a molecule that is essential for cells of the target tissues, but cannot be synthesized by the cells themselves. Vitamins meet these criteria, and vitamin E in particular is the most promising of the fat-soluble vitamins due to its low toxicity, even at high doses^4^. Recently, we directly conjugated α-tocopherol, a major natural isomer of vitamin E, to siRNA (termed Toc-siRNA) and observed a substantial reduction in *Apolipoprotein B* (*ApoB*) gene expression in the mouse liver following intravenous injection, which was achieved due to the binding with serum lipoproteins, including high-density lipoprotein^5^. Nevertheless, although the efficiency of delivery was relatively high, lipid-conjugated-siRNA was diffusely distributed to non-target organs as well^6^. Dietary vitamin E is absorbed in the jejunum and incorporated into the chylomicrons assembled in the mucosal epithelial cells; subsequently, these chylomicron-vitamin E complexes are transported to the systemic circulation via the lymphatic system. Serum lipoprotein lipase converts the chylomicrons to chylomicron remnants, and these remnants are selectively and rapidly taken up by hepatocytes via remnant receptors^7^. Hence, postprandial enteral delivery of Toc-siRNA may help achieve the specificity of siRNA delivery to the liver due to the intrinsic lipid transport system via chylomicron.

Hyperlipidemia is regarded as one of the major risk factors for coronary heart disease (CHD) and ischemic stroke, both of which are leading causes of death in many countries. Although serum low-density lipoprotein (LDL) cholesterol levels are lowered by 3-hydroxy-3-methylglutaryl coenzyme A reductase inhibitors (statins), such medicaments are not sufficiently efficacious in approximately one-third of hyperlipidemic patients for achievement of acceptable LDL cholesterol levels; in fact, only 30% of patients with very-high-risk CHD experience success with these therapies^8^,^9^. Thus, establishment of an additional therapeutic technology to prevent CHD is an urgent clinical need. In this context, many groups have tried to address this issue with silencing of the *ApoB* gene, which is relevant to blood LDL cholesterol and triglyceride levels. Effective systemic delivery of siRNA has been achieved only through intravenous injection, which considerably limits its practical applications for such life-style-related diseases because of the need for medical support and the risk of adverse events such as infection and shock. Therefore, to expand the clinical applications of siRNA technologies, it is necessary to develop an enteral delivery system. However, conventional oral dosage forms are not applicable to polar macromolecules such as siRNA because of its poor absorption across the gastrointestinal epithelium and instability against ribonucleases. Indeed, there are few examples of techniques for intestinal delivery of nucleic acids, a prerequisite for a practical preparation, even at the level of research on laboratory animals. Success in achievement of anti-inflammatory action with oral administration of siRNA in a particulate delivery system has been reported^10^. However, this study was based on delivery of siRNA to the M cells of Peyer’s patches, which are immune tissues in the gastrointestinal tract, and macrophages mediated siRNA transport across the gastrointestinal epithelium should be limited.

Here, we describe a novel technique for intestinal oligonucleotide delivery that included mucosal penetration with an absorbefacient and *in vivo* fabrication of a drug delivery system (DDS) with an endogenous carrier in the lymphatic; this enabled enteral and hepatocyte-specific siRNA delivery and therapeutic gene silencing, leading to “oral RNAi therapy.”

## Results

### Formulation of Toc-siRNA in LNPs

A phosphoramidite was prepared using the hydroxyl group at the C6 position of α-tocopherol and bound directly to the 5′-end of the antisense strand of a 29-base siRNA molecule^5^,^11^ that was chemically modified to selectively silence *ApoB* expression in the liver. A sense strand with 27 corresponding bases was bound to a fluorochrome (Cyanine 3, Cy3) for tracking and annealed to produce fluorescently labeled Toc-siRNA. The size distribution of Toc-siRNA *per se* varied among preparations. Dynamic light-scattering (DLS) analysis suggested that Toc-siRNA formed self-associated micelles and nano-aggregates, likely due to its amphipathic properties (Supplementary Fig. S1). The peak diameter of the Toc-siRNA micelles was approximately 10 nm. Toc-siRNA were efficiently incorporated into the mixed micelles (MM) that comprised linoleic acid and PEG-60 hydrogenated castor oil (HCO-60), to form LNPs having a single peak distribution (polydispersity index, 0.103) with the mean diameter of approximately 15 nm (Supplementary Table S1 and Supplementary Fig. S1). Filtration was needed for preparing nano-sized monodisperse MM, because some submicro- or micro-aggregates or agglomerates were occasionally observed without filtration (Supplementary Fig. S2). Consequently, we could formulate Toc-siRNA as a fine LNP with linoleic acid and HCO-60.

### Hepatic delivery of Toc-siRNA by the LNPs

**Figure 1:**
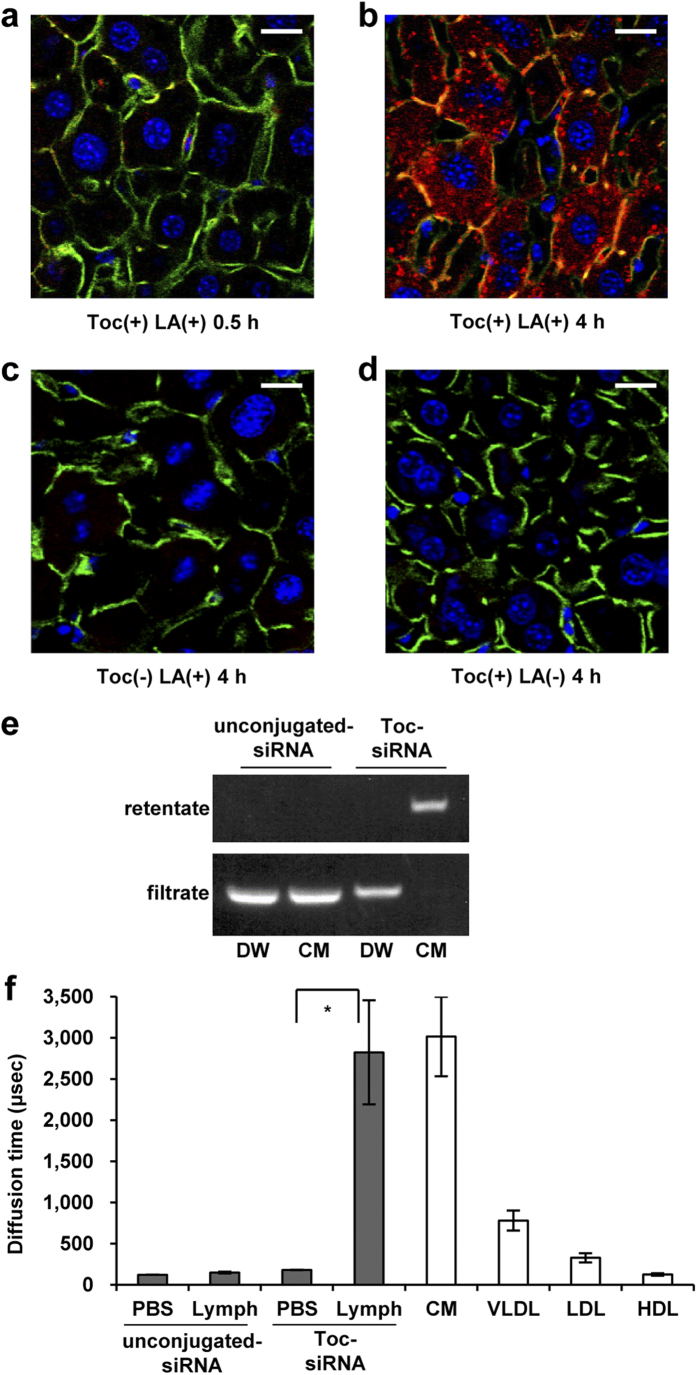
Enteral delivery of Toc small interfering RNA (siRNA) to the mouse liver. (**a,b**) Confocal images of mouse livers at 0.5 h (**a**) and 4 h (**b**) after administration of lipid nanoparticles (LNP) to the colorectum. (**c,d**) Four hours after administration of LNP to the colorectum without α-tocopherol conjugation (**c**) and without linoleic acid (**d**) as controls. The dose of Toc-siRNA was 10 mg/kg body weight. Red: Cy3-labeled Toc-siRNA; Green: FITC-Phalloidin, which labels the cytoskeleton; Blue: TO-PRO-3, which labels cell nuclei. Toc: Cy3-labeled Toc-siRNA; LA: linoleic acid. Bar = 20 μm. (**e**) Gel-shift assay of filtered Toc-siRNA incubated with chylomicron (CM) extracted from the lymph. DW, distilled water. (**f**) Fluorescence correlation spectroscopy (FCS) analyses (diffusion time) of Cy3-labeled Toc-siRNA in lymph extracted from mice 2 h after administration of LNP to the colorectum and from mice administered LNP without an α-tocopherol moiety as controls. *n* = 10, mean values ± s.e.m.; *P < 0.05.

First, we evaluated the effects of LNPs on enteral delivery of siRNA to the liver in mice under postprandial conditions. A single dose of LNPs (10 mg/kg of body weight as Toc-siRNA) was administered to the jejunal loop where orally ingested α-tocopherol is normally absorbed, revealing almost no Cy3 fluorescence in the liver 4 h after dosing (Supplementary Fig. S3). In contrast, when LNPs were administered to the colorectal loop, delivery of the siRNA into the liver was observed in a time-dependent manner (Fig. 1a,b and Supplementary Fig. S4); robust dot-like Cy3 signals were proven to localize in the cytoplasm of most hepatocytes and non-parenchymal cells in liver sinusoids 4 h after administration. The micro-sized LNP particles might interfere with the hepatic delivery of Toc-siRNA in nano-sized LNP particles, because the hepatic delivery of Toc-siRNA due to LNPs was revealed to be enhanced with the filtration (Supplementary Fig. S4).

This delivery of siRNA into hepatocytes was markedly reduced when siRNA was not bound to α-tocopherol (Fig. 1c) or when the absorbefacient was absent (Fig. 1d), indicating that both the absorbefacient and the conjugation with α-tocopherol were essential to this delivery system. Actually, we confirmed *ex vivo* by gel-shift assay that Toc-siRNA efficiently associated with the chylomicron, but not without α-tocopherol conjugation (Fig. 1e). Furthermore, the measured diffusion time on fluorescence correlation spectroscopy (FCS) analysis, which reflects the size of the particle carrying a fluorescent signal, also indicated *ex vivo* that Toc-siRNA efficiently associated with the lymph, most likely with the chylomicron fraction (Fig. 1f); the diffusion time of the Cy3 signal in the lymph was approximately 3,000 μsec and corresponded to that of chylomicrons, which were obtained through fractionation by FPLC. The function of α-tocopherol is likely to couple hydrophilic siRNA to chylomicrons because of its hydrophobicity.

**Figure 2:**
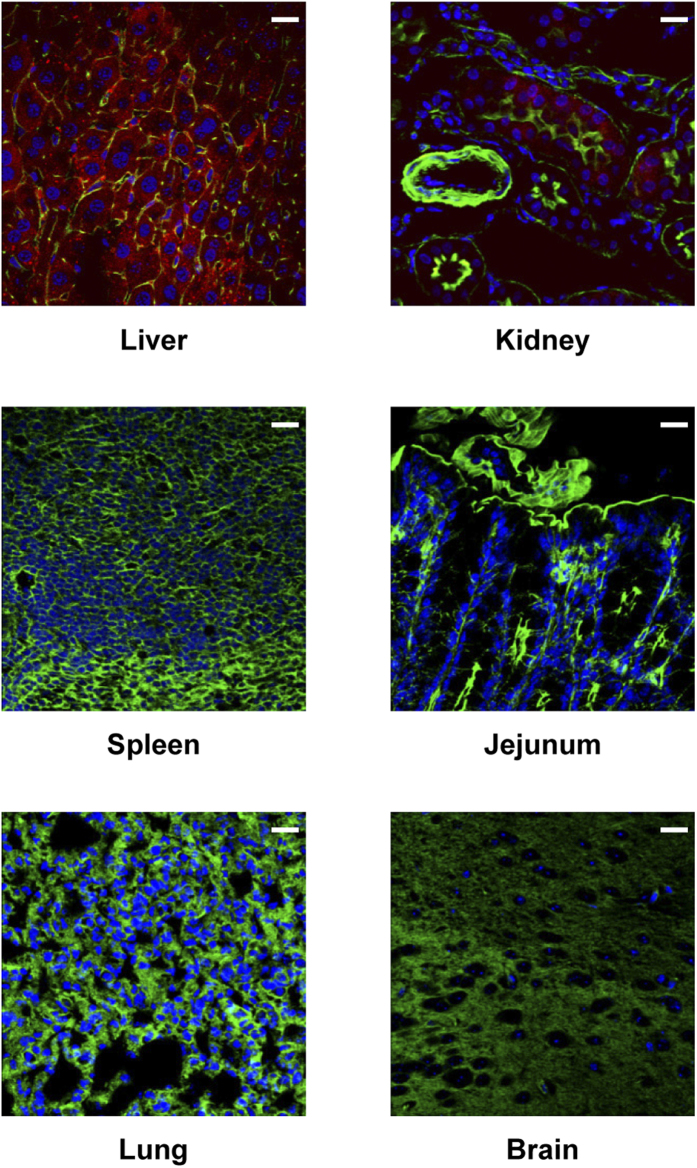
Fluorescence micrographs of various mouse organs 4 h after colorectal administration of LNP. Red: Cy3-labeled Toc-siRNA, Green: FITC-Phalloidin, Blue: TO-PRO-3, Bar = 20 μm.

With respect to biodistribution, the majority of the Cy3 fluorescence was found in the liver by the confocal microscopy (Fig. 2), although a low level was also detected in the kidney, indicating that this delivery system promoted targeting to the liver. This was supported by a quantitative analysis of the tissue distribution of fluorescent signals, although a low level was detected not only in the kidney, but also in the intestine (Supplementary Fig. S5). The distribution in the kidney and intestine may reflect the disposition or excretion of Toc-siRNA. The liver Toc-siRNA level after rectal administration was approximately ten times lower than the level observed after intravenous injection (Supplementary Table S2); however, in a comparison of the ratios of liver to serum concentrations of Toc-siRNA, rectal administration showed a much higher value than intravenous injection, suggesting that the enteral route provides more efficient delivery of siRNA to the liver than the parenteral route.

### *In vivo* gene silencing effect by the LNPs

**Figure 3:**
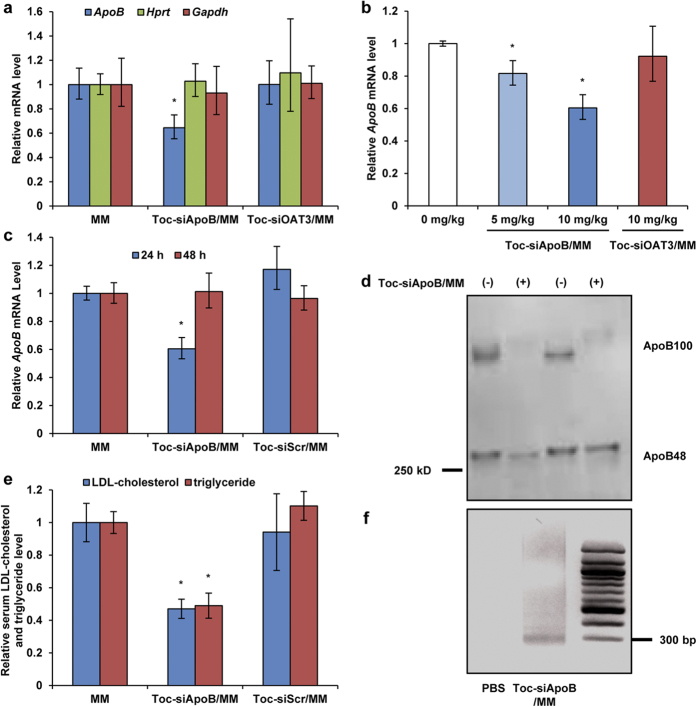
Ability of LNP to silence target gene in the mouse liver. LNP (with Toc-siRNA including Toc-siApoB targeting to mouse *ApoB* mRNA, Toc-siOAT3 targeting to mouse *Oat3* mRNA, or Toc-siScr scrambled sequence of Toc-siApoB) or MM (without Toc-siRNA) was rectally administered as enema to the mice at a dose of 10 mg/kg of Toc-siRNA three times with 2-h intervals. (**a**) Quantitative RT-PCR analyses of endogenous *ApoB*, *Gapdh*, and *Hprt* mRNA levels were performed 24 h after final administration. (**b**) Dose-dependent reduction of *ApoB* mRNA levels in the liver after administration of different doses of LNP. The *ApoB* mRNA levels (normalized to *Gapdh* mRNA levels) were determined 24 h after final administration. (**c**) Quantitative RT-PCR analysis of liver *ApoB* mRNA levels relative to *Gapdh* mRNA performed at the indicated time points after final administration. (**d,e**) Western blot analysis to detect ApoB100 and ApoB48 (**d**), and LDL-cholesterol and triglyceride levels in serum (**e**) 24 h after final administration. All experiments were independently performed in duplicate. The quantitative data were normalized to administration of mock mixed micelles alone (MM) and shown as the mean values ± s.e.m. (*n* = 3). *P < 0.05. (**f**) Agarose gel of 5′-RACE–PCR amplification, showing specific cleavage products in the liver.

Next, we evaluated the therapeutic efficacy of Toc-siRNA by enteral delivery in mice. The levels of endogenous *ApoB* mRNA in the liver were measured in order to assess the *in vivo* silencing ability of Toc-siRNA. Non-labelled Toc-siRNA was rectally administered as LNP containing linoleic acid 3 times a day at a dose of 10 mg/kg (i.e., total 30 mg/kg), and *ApoB* mRNA expression was examined in hepatocytes using quantitative RT-PCR. *ApoB* mRNA expression was significantly suppressed by approximately 40% (Fig. 3a); this silencing effect was dose-dependent (Fig. 3b) and returned to baseline levels 48 h after administration (Fig. 3c). In addition, corresponding to a remarkable reduction in serum ApoB100 protein levels (Fig. 3d), both serum LDL-cholesterol and triglyceride levels were found to decrease significantly, by approximately 40%, after 24 h (Fig. 3e). In contrast, such reductions in target gene expression and serum ApoB100 and lipid levels were not observed in mice that were rectally dosed with α-tocopherol-conjugated siRNAs with a scrambled or unrelated sequence with respect to *ApoB* (Fig. 3a–c,e).

In order to examine that the *in vivo* activity was due to siRNA-directed cleavage, we determined specific mRNA cleavage site using a modified 5′-RACE (rapid amplification of cDNA ends) technique^1^. The amplified cleaved PCR fragment was same as that reported in liver of animals receiving cholesterol-conjugated siRNA targeting the same mouse *ApoB* mRNA^1^ (Fig. 3f), indicating the predicted cleavage in the *ApoB* open reading frame by siRNA.

On the other hand, off-target effects on unrelated genes in the liver were not noted (Fig. 3a). No remarkable toxic or immune-stimulatory side effects were observed through blood biochemical analyses (Table 1). Moreover, histological analysis did not show pronounced local mucosal irritation with LNP (Supplementary Fig. S6). Hence, we demonstrated that this enteral delivery system enabled therapeutic gene silencing in the liver without obvious adverse reactions.

### *In vivo* Toc-siRNA delivery via the lymphatic route

**Figure 4:**
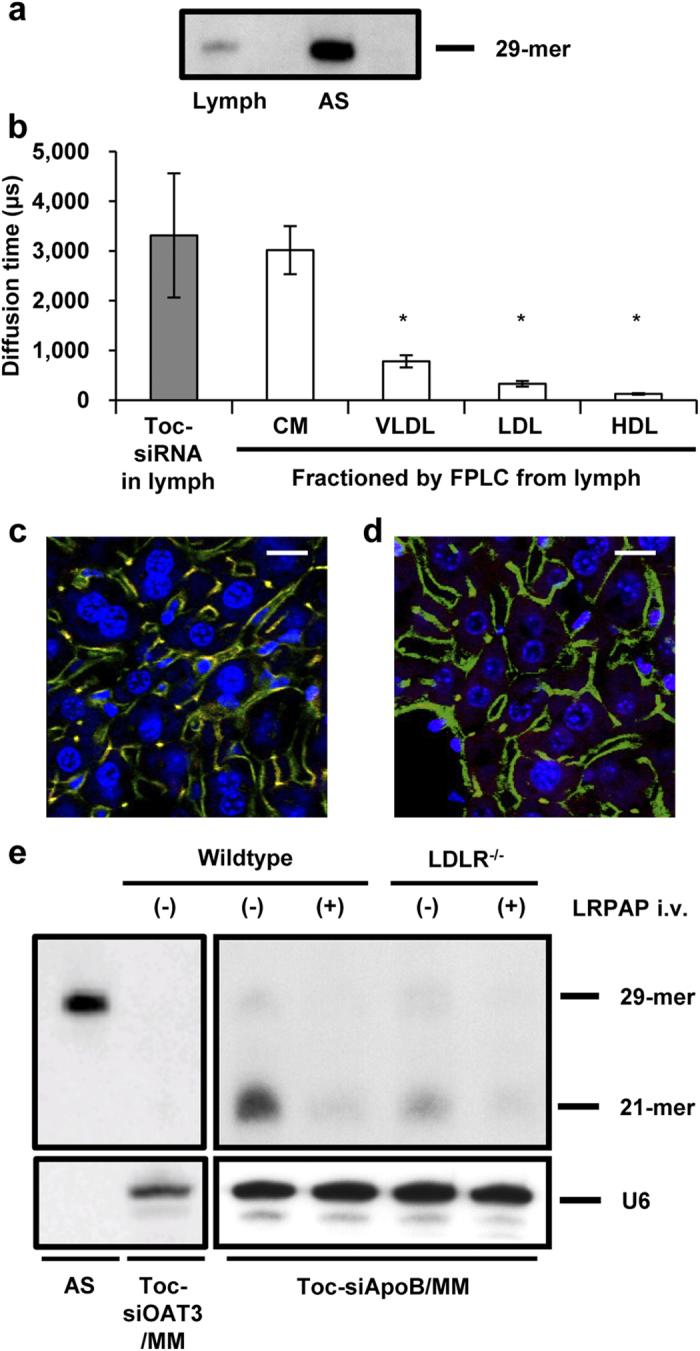
Delivery of Toc-siRNA into liver. (**a**) Northern blot analysis to detect Toc-siRNA antisense strand in lymph from mouse 2 h after administrated by LNP with Toc-siRNA. AS: Antisense strand of Toc-siRNA. (**b**) FCS analyses (diffusion times) of Cy3-labeled Toc-siRNA in lymph extracted from mice 2 h after colorectal administration and of Nile Red-labeled, FPLC-fractionated lipoproteins prepared from untreated mice. CM, chylomicron; VLDL, very-low-density lipoprotein; LDL, low-density lipoprotein; HDL, high-density lipoprotein. *n* = 3, mean values ± s.e.m; *P < 0.05. (**c,d**) Confocal images of mouse livers 4 h after colorectal administration of LNP in the fasting state (**c**) or after feeding of milk and intravenous injection of an LPL inhibitor 30 min prior to dosing (**d**). Red: Cy3-labeled Toc-siRNA, Green: FITC-Phalloidin, Blue: TO-PRO-3, Bar = 20 μm. (**e**) Northern blot analysis to detect Toc-siRNA antisense strands in the livers from LNP (with Toc-siRNA) administrated wildtype or LRPAP-injected LDLR^-/-^ mouse. U6 was used as a loading control. AS: Antisense strand of Toc-siRNA.

We then investigated the mechanisms for this enteral delivery. The antisense strand of Toc-siRNA was detected by northern blot analysis in the lymph that was collected from the lymphatic vessel after administration of LNP with Toc-siRNA to the colorectal loop in the mice (Fig. 4a). Next, we measured diffusion time on FCS of the same *in vivo* lymph. The diffusion time of the Cy3 signal in the lymph corresponded to that of chylomicrons (Fig. 4b), indicating that the particle carrying the Toc-siRNA was chylomicrons in lymph. Chylomicron is secreted into the lymphatics along with fat absorption and rarely observed under fasting state. As we expected, Cy3 fluorescence was hardly detected in the liver in the fasting state (Fig. 4c). These results suggest that Toc-siRNA absorbed from colorectal tract bounds to chylomicron to form a complex in the lymph at the jejunum or thoracic level while ascending in lymph ducts. Additionally, because chylomicrons are converted to remnants through lipolysis by lipoprotein lipase and become capable of binding to remnant receptors of hepatocytes^12^, we next examined the effects of a lipoprotein lipase inhibitor on the accumulation of Cy3 signals in the liver. Intravenous administration of Triton WR1339^13^ prior to intestinal dosing with Toc-siRNA LNP significantly reduced Cy3 signals in hepatocytes despite examination during postprandial state (Fig. 4d). Taken together, these results indicated that both chylomicrons and lipoprotein lipase were indispensable for the transfer of Toc-siRNA into hepatocytes.

### Cellular uptake of Toc-siRNA by hepatocytes

Finally, we examined whether this delivery of Toc-siRNA into hepatocytes was mediated by remnant receptors; the LDL receptor (LDLR) and LDL receptor-related protein 1 (LRP-1)^12^. Using northern blot analysis, we detected much less siRNA in the livers of LDLR^–/–^ mice and almost none in LDLR^–/–^ mice injected with LDL receptor-related protein-associated protein 1 (LRPAP) (Fig. 4e), which inhibits the ligand-binding ability of the remnant receptors^14^. In addition, capitalizing on the activity of Toc-siRNA would require transfer of large target molecules from endosomes to the cytoplasm and activation via cleaving enzymes such as Dicer. The northern blotting was performed with a probe to label the antisense strand of Toc-siRNA, revealing 2 antisense strands that corresponded to the 29 mers in the strand originally administered and to the 21 mers strand length indicative of cleavage by Dicer in cells (Fig. 4e). This indicated that Toc-siRNA taken up by mouse hepatocytes reached the cytoplasm and was converted into the mature form of 21-mer siRNA. Thus, these results indicated that efficient delivery of Toc-siRNA to the liver requires formation of a complex with chylomicrons in the lymph, conversion of chylomicrons to remnants by lipoprotein lipase, and remnant receptor-mediated uptake by hepatocytes.

## Discussion

**Figure 5:**
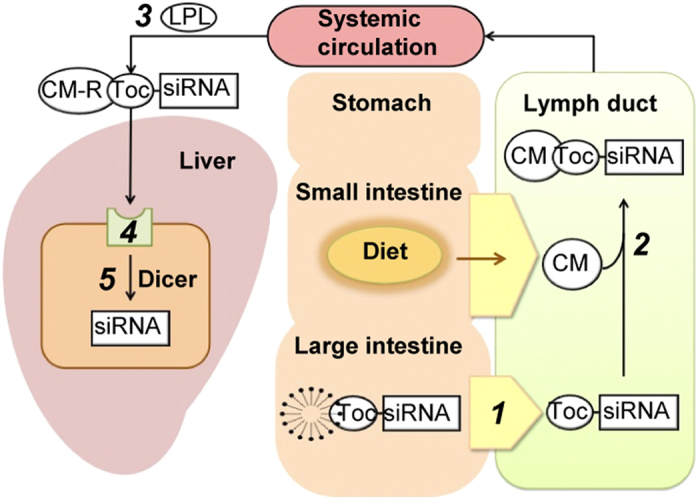
Schematic diagram of hepatocyte-specific delivery of Toc-siRNA to hepatocytes via an intestinal route and *in vivo* fabrication. Postprandial chylomicrons (CM), accompanying the absorption of lipids from the diet, are produced in the epithelium of the small intestine and secreted into the lymphatic system. ***1**)* The LNP formulation and an absorbefacient allow Toc-siRNA to penetrate the colonic mucosa and be transferred into the lymphatic system. ***2**)* Incubation in the lymphatic vessels allows Toc-siRNA and CMs to form complexes (i.e., *in vivo* fabrication). ***3**)* CMs are converted to CM remnants (CM-Rs) by lipoprotein lipase (LPL) in the systemic circulation. ***4**)* Toc-siRNA-CM-R complexes are selectively and efficiently taken up by hepatocytes through transport via remnant receptors. ***5**)* Toc-siRNA is processed by Dicer to produce active form of 21-mer siRNA.

Although synthetic functional nucleic acid molecules that are highly active and techniques for molecular design and synthesis are being developed, little progress has been made in the development of techniques to safely and efficiently deliver nucleic acid molecules to target organs and cells. siRNA is a double-stranded oligonucleic acid, 21–23 bases in length with a molecular size of approximately 13 kD or larger. Penetration of cell membranes and epithelial tissues by such hydrophilic macromolecules is seldom seen physiologically except when special transport systems, such as endocytosis and transcytosis, are active^15^. The development of non-parenteral conventional preparations, such as oral formulations or suppositories, is very challenging in RNAi-based therapeutics. Here, we designed an enteral siRNA delivery system based on the proposed concept of *in vivo* fabrication where the carrier complex of siRNA targeting hepatocytes is ultimately assembled in lymphatic by recruiting chylomicrons. This new technique of our enteral siRNA delivery system comprises three major processes as illustrated in Fig. 5: (1) intestinal mucosal penetration, (2) lymphatic transfer and fabrication with chylomicrons through *in vivo* incubation, and (3) receptor-mediated uptake by hepatocytes. The first step is related to the stability and permeability of the formulated siRNA, while the latter two steps are related to targeting via lymphatic transport in a postprandial condition.

To overcome the first hurdle, we synthesized a highly ribonuclease-resistant siRNA with 2′-*O*-methyl and phosphorothioate internucleotide linkage modifications, followed by conjugation to α-tocopherol and formulated it as an LNP with linoleic acid as an absorbefacient and HCO-60 as an emulsifier. The hydrophobic property of the α-tocopherol moiety allowed Toc-siRNA to bind to LNPs, thus preventing self-assembly of Toc-siRNAs. α-tocopherol is known to have a high affinity for linoleic acid^16^; this may contribute to facilitate the formulation of LNP. Additionally, electrostatic repulsion due to the highly negative charge of siRNA was expected to suppress formation of larger aggregates, thereby stabilizing the LNPs, as suggested by DLS (Supplementary Fig. S2). Formulation of enteral drugs as nanoparticles has been reported to enhance intestinal drug absorption via beneficial effects on aqueous dispersion and access to intestinal epithelium. Lipophilic compounds, such as fatty acids and cholesterol, are known to be largely diffusion-limited through the unstirred water layer overlying the luminal surface of the intestinal tract. Indeed, Toc-siRNAs incorporated into the nano-emulsion were more efficiently delivered to the target liver compared to those incorporated into mixtures of nano- and micro-emulsions (Supplementary Fig. S4).

The physiological site of absorption for vitamin E is the small intestine, chiefly the jejunum, but administration of LNPs to the jejunum resulted in the lowest delivery of Toc-siRNA to the liver (Supplementary Fig. S3). This is conceivably due to the poor permeability of hydrophilic macromolecules, such as siRNA, through the plasma membranes of enterocytes^15^. Then, we changed our strategy by taking advantage of the large intestine as the site of absorption, where paracellular transport of siRNA via transient opening of tight junctions between colorectal enterocytes with the help of an absorption promoter is easier than in the jejunum^17^. Various molecules have been reported to act as absorption enhancers, including fatty acids, bile salts, surfactants, chelating agents, and new classes of tight-junction modulators^17^,^18^. Among these, natural lipids are promising adjuvant candidates for clinical use because of their high bioavailability and biocompatibility. Of the natural lipids, linoleic acid was reported to be one of the most effective absorption enhancer for enteral delivery^17^. Therefore, we selected linoleic acid as an absorbefacient. Indeed, Toc-siRNA can be absorbed in the colorectum much more efficiently than in the jejunum when it was administered as LNP with linoleic acid. This site-dependent difference is probably due to a much higher clearance of fatty acids in enterocytes in the jejunum than in the colorectum^19^. In addition, the small intestine naturally has greater luminal fluid secretion or content than the large intestine, which would lead to a decrease of fatty acid concentrations in the lumen or the epithelial surface vicinity, thereby reducing in their effects.

Epithelial barrier function is essentially dependent on the integrity and contribution of cytoskeletal and membrane proteins that comprise and regulate tight-junction complexes existing between enterocytes. Many of those key proteins are known to be sulfhydryl proteins^20^,^21^. The mechanisms underlying the transient increase in mucosal permeability produced by fatty acids remain to be fully elucidated, but enhanced mucosal permeability by unsaturated long-chain fatty acids may be regulated not only by membrane fluidity, but also by the redox state of sulfhydryl proteins^22^. It is probable that linoleic acid increases paracellular permeability by affecting tight junction complexes and the perijunctional actomyosin ring via the induction of intracellular acidification and increased intracellular Ca^2+^ levels^23^. Changes in the physical state of the lipid raft of the apical membrane of enterocytes may also be involved in the mechanism since fatty acids incorporated into the lipid raft can increase the membrane fluidity^17^.

We hypothesized that chylomicrons in the lymph could act as an *in vivo* vehicle for postprandial lipid-mediated delivery of Toc-siRNA to the liver. Hence, lymphatic transfer was the essential process required for this purpose. The selectivity of transfer processes between blood and lymph circulation after passing through the colorectal epithelium is likely based on a simple molecular sieving mechanism in which molecular size is a major determinant. The molecular size threshold of transport from the rat jejunum and colorectum to the lymph rather than to the blood is reported to be approximately 18–39 kD and 10–18 kD, respectively^24^,^25^. Our designed Toc-siRNA, with a molecular weight of approximately 19 kD, was anticipated to undergo lymphotropic transfer in the colorectal mucosa. Lipophilic modification with α-tocopherol may also facilitate transfer of siRNA molecules into lymphatic vessels due to increase of apparent molecular size possibly via protein binding or self-association. In addition, interstitial hydration and increased lymph formation caused by linoleic acid may contribute to this preferable transfer to lymphatic vessels by increasing the convective movement of interstitial fluid toward lymphatic vessels^26^. Indeed, Toc-siRNA delivery to the liver was not observed under the fasting conditions (Fig. 4c), suggesting that direct hepatic transfer via the portal vein from the intestine, if any, is not a major pathway.

A critical step in our delivery system for Toc-siRNA is to undergo “*in vivo* incubation” with the lymph in the lymphatic vessel, where endogenous chylomicrons are excreted at a high level. This mechanism permits more efficient binding of Toc-siRNA to chylomicrons to form a complex as an *in vivo* DDS, resulting in more specific delivery to the liver. Serum chylomicron content is low even under postprandial condition because of its rapid metabolism by lipoprotein lipase. Most of the Toc-siRNA administered intravenously was hence incorporated into other lipoproteins or serum proteins, such as HDL or albumin^5^. In contrast, chylomicron levels in the intestinal lymphatic duct increase much after meals or by feeding fat. In the lymph from Toc-siRNA-administered mouse, we directly confirmed the existence of siRNA on northern blot analysis (Fig. 4a) and demonstrated the binding of siRNA to chylomicron by FCS (Fig. 4b). Hydrophobic property of α-tocopherol enabled this efficient binding of Toc-siRNA to chylomicrons by “*in vivo* incubation” in the intestinal lymphatic duct. Chylomicron, as an endogenous vector, may work in not only increasing its delivery efficiency to the liver, but also achieving more preferentially targeted delivery of siRNA to the liver than injection methods (Supplementary Table S2). Generally, targeting cell- or organ-specific delivery of siRNA is necessary to avoid side effects caused by siRNA distributing to undesirable sites. The relatively specific distribution of Toc-siRNA to the liver suggested that it was preferentially transferred to the lymph rather than the blood circulation since LDL and HDL receptors are distributed widely throughout the body^6^. Indeed, when administered intravenously, cholesterol-conjugated siRNA is delivered into many organs by binding to serum HDL^27^. Thus, it was suggested that the uptake of Toc-siRNA via the HDL receptor is not the major mechanism of its hepatic delivery because Toc-siRNA was hardly detected in the LDLR^–/–^ mice which was administered the LRPAP inhibitor although the transport by HDL receptor is still active (Fig. 4e). Taken together, these data indicate that the *in vivo* fabrication of Toc-siRNA and chylomicron complex can be used to achieve efficient and specific gene silencing in the liver.

In the current study, to establish proof of concept of enteral delivery, we applied high doses of siRNA (i.e., 10–30 mg/kg), which is not very practical in clinical applications. Further studies are required to increase the efficacy of the enteral delivery system. This should be achieved by optimization of the formulation of the LNP as well as the active ingredients or nucleic acids. We previously reported the use of polymeric materials to facilitate endosomal escape via the acidification process of endolysosomes (e.g., the proton-sponge hypothesis)^28^,^29^. In the present study, the intracellular disposition in the liver was not fully examined because northern blotting demonstrated a relatively efficient cleavage of Toc-siRNA by Dicer, which exists in the cytosol. The efficacy of gene silencing by the enteral delivery system may be further improved by adding an endosomal escape mechanism. *N*-Acetylgalactosamine (GalNAc) is a highly efficient ligand for the asialoglycoprotein receptor in hepatocytes^30^,^31^,^32^; accordingly, GalNAc conjugation may conceivably improve the specific delivery of siRNA to hepatocytes. We have recently developed new oligonucleotide types, such as α-tocopherol-conjugated chimeric antisense oligonucleotides^33^ or DNA/RNA heteroduplex oligonucleotides^34^, which show much higher gene silencing effects. However, even with higher doses of administration, enteral dosage forms provide much higher patient acceptance and long-term compliance, which are required in therapies for chronic diseases, such as lifestyle-related diseases like hyperlipidemia and for primary and secondary prevention of atherosclerotic vascular diseases, including CHD and ischemic stroke. Furthermore, compared to the parenteral route, a higher targeting efficiency can be obtained via enteral delivery, which has advantages in terms of safety as well as efficacy.

Long-term stability is a major practical issue for liquid dosage forms, such as enemas, especially those comprising emulsions like LNP. LNP can be incorporated into a suppository following lyophilization and may also be formulated as an oral dosage form, i.e., enteric-coated preparations, or by integration with a colon-targeting delivery technique. These dosage forms enable repeated, simpler administration of siRNA than injections, opening the possibility of their application not only to prevent atherosclerotic vascular diseases, but also to combat common chronic liver diseases, such as viral hepatitis and liver cancer. In addition, optimization of the formulation of the enteral delivery system should probably allow reducing the therapeutic doses. On the other hand, the long-term effects and potential side effects of the delivery system remain to be clarified.

In summary, we have developed a novel technology for enteral delivery of siRNA to achieve effective gene silencing in the liver. To the best of our knowledge, this is the first report of successful liver-specific gene silencing based on truly systemic RNAi via enteral delivery in mice. We consider that this may define a basic platform for enteral delivery of nucleic acid drugs to the liver.

## Methods

### Synthesis of siRNA

siRNAs were chemically synthesized by Hokkaido System Science (Sapporo, Japan). To combine α-tocopherol and siRNAs, *dl*-α-tocopherol (Tokyo Kasei, Tokyo, Japan) was *O*-phosphitylated with 2-cyanoethyl-*N,N*-diisopropylphosphoramidite and diisopropylethylamine in tetrahydrofuran, yielding α-tocopherol phosphoramidite, followed by coupling with the 5′ end of the antisense siRNA^5^,^11^. Synthetic sense and antisense siRNA strands were then annealed. The sequences of Toc-siRNA are presented in Supplementary Table S3.

### Animal experiments

Eight-week-old imprinting control region (ICR) mice (CLEA Japan, Tokyo, Japan), 7-week-old C57BL/6J mice (Oriental Yeast, Tokyo, Japan), and 7-week-old B6.129S7-Ldlr (tm1Her)/J mice (Jackson Laboratory, ME) were used. An *in situ* intestinal loop technique was employed. Briefly, mice were fasted for 16 h and then fed 0.3 ml condensed milk containing 20% fat 3 times at 30-min intervals. At 30 min after the last feeding of milk, mice were anesthetized with 40 mg/kg sodium pentobarbital (Nembutal, i.p.; Dainippon Sumitomo Pharma, Osaka, Japan). A 5-cm intestinal loop was then made in the jejunum or colorectum by placing a silicon catheter into the proximal side of the loop. The loop was washed 3 times with 2 ml saline (37 °C), followed by flushing with syringe air to remove any remaining saline, and the distal side or anus was then ligated with a silk thread or metal clip, respectively. Cy3-labeled Toc-siRNA, free or as LNPs or concomitantly with other absorption enhancers, was administered (total volume, 5 μl per gram of body weight) at the dose of 10 mg/kg. Mice were sacrificed by replacement of their blood with cold saline (Otsuka Pharmaceutical, Tokushima, Japan) at 0.5 or 4 h after Toc-siRNA administration in single-dose experiments, or at 2, 24, or 48 h after the last administration in triple-dose experiments, wherein Toc-siRNA was administered 3 times at 2-h intervals. Organs were excised for confocal imaging, western blot analysis, northern blot analysis, 5′-RACE analysis, and quantitative RT-PCR (qRT-PCR). For FPLC and FCS analyses, lymph samples were collected through a vinyl catheter (SV 31, Natsume Seisakusho, Tokyo, Japan) placed in the thoracic lymphatic duct for over 2 h after the colorectal administration of the test solution. In the LDLR^–/–^ mouse study, Toc-siRNA was colorectally administered immediately after injection of 0.8 mg/kg recombinant mouse LRPAP (R&D Systems, MN) via the tail vein. For the study of LPL involvement, 500 mg/kg Triton WR 1399 (Tyloxapol, Sigma, MO), an LPL inhibitor, was injected into the tail vein 30 min before dosing with Toc-siRNA.

Total RNA for qRT-PCR was isolated from the liver using Isogen (Nippon Gene, Tokyo, Japan), and small RNA was isolated using MirVana (Ambion, TX) for northern blot analysis. Blood samples were collected at 2 h after the final administration to determine interferon (IFN)-α levels using the Mouse IFN-α ELISA Kit (PBL Biomedical Laboratories, NJ) and at 24 h after the final administration for western blotting analysis and measurement of LDL-cholesterol, triglycerides, creatinine, alanine aminotransferase, sodium, and potassium.

For histopathological analysis, the colon was postfixed in 4% paraformaldehyde (Wako Pure Chemical Industries, Osaka, Japan) for 6 h, embedded in paraffin, sectioned (14-μm-thick sections) using a Leica RM 2235 Microtome (Leica Microsystems, Wetzlar, Germany), and then stained with hematoxylin and eosin.

Animal use was according to National Institutes of Health guidelines, and the care and treatment of all animals was in strict accordance with protocols approved by the Institutional Animal Experiment Committees of Osaka Ohtani University (#11-01) and Tokyo Medical and Dental University (#0130113A).

### Preparations for *in vivo* enteral siRNA delivery

A mixture of PEG-60 hydrogenated castor oil (HCO-60; Nikko Chemicals, Tokyo, Japan) and linoleic acid (*cis, cis*-9,12-octadecadienoic acid, Wako Pure Chemical Industries), 4,7,10,13,16,19-docosahexaenoic acid (DHA), or 5,8,11,14,17-eicosapentaenoic acid (EPA, Cayman Chemicals, MI) was sonicated in RNase-free PBS (pH 7.4) for 5 min on ice using a Digital Sonifier Models S-250D (Branson, CT) at 20 kHz and 30 W. Mixed micelles were further filtered through a membrane filter (pore size, 0.45 μm; Millipore, MA). Before administration, Cy3-labeled siRNA, Cy3-labeled Toc-siRNA, or Toc-siRNA was added to the mixed micelles. Final concentrations of fatty acids and HCO-60 were 10 mM and 3.0% (w/w), respectively. Formation of LNPs was confirmed by dynamic light scattering (DLS). The following absorption promoters were also used in the aqueous solution with Cy3-labeled Toc-siRNA: 15 mM sodium caprate (Sigma-Aldrich, MO); 20 mM citric acid (NacalaiTesque Co. Ltd., Kyoto, Japan); 2% (w/v) Labrasol, a solubilizer and bioavailability enhancer consisting of caprylocaproyl macrogol-8-glycerides (Gattefossé, Nanterre Cedex, France); and 10 mM lauryl maltoside (n-dodecyl-β-D-maltoside, Sigma-Aldrich, MO).

### Confocal imaging

Organs were fixed with 4% paraformaldehyde solution overnight, followed by incubation in 30% sucrose in PBS (Wako Pure Chemical Industries) overnight. Samples were then mounted in O.C.T. Compound (Sakura Finetek, Tokyo, Japan). Frozen sections were made from each sample using a Cryostat (Leica Microsystems) and stained with TO-PRO-3 (a carbocyanine monomer nucleic acid stain with far-red fluorescence from Invitrogen, CA) and Alexa-488 Phalloidin (Invitrogen). Confocal images were taken with an LSM510 META (Carl Zeiss Microscopy, Jena, Germany).

### Gel-shift assay

Toc-siRNA (500 pmol) were added to 25 μl of chylomicron fraction from mouse lymph or distilled water. The samples were filtered using Microcon YM-100 (Millipore). Both retentates and filtrates were resolved by electrophoresis on a 2% agalose gel for 5 min at 100 V. The oligonucleotides were visualized under UV after staining the gel with ethidium bromide in Tris-borate-EDTA buffer.

### Fluorescence correlation spectroscopy (FCS) analysis

Measurements were performed using the ConfoCor 3 module in combination with an LSM 510 system (Carl Zeiss Microscopy) equipped with a C-Apochromat 40 × /1.2W objective. An He-Ne laser (543 nm) was used for Cy3-labeled Toc-siRNA excitation in lymph or the lipoprotein fraction of lymph labeled with Nile Red (Tokyo Chemical Industry, Tokyo, Japan). Emissions were filtered through a 560–615 nm band-pass filter. Samples were placed into 8-well Lab-Tek chamber slides (Nalgene Nunc International, NY), and diffusion time was measured at room temperature. Autocorrelation curves obtained from 10 measurements with a sampling time of 20 s were fitted using ConfoCor 3 software to determine diffusion times.

### Quantitative RT-PCR

Total RNA was reverse transcribed with Superscript III and random hexamers (Invitrogen). qRT-PCR was performed on 2 μg of cDNA with LightCycler 480 Probes Master (Roche Diagnostics, Basel, Switzerland) following the manufacturer’s instructions. Amplification conditions were 40 cycles of 95 °C for 15 s, 60 °C for 30 s, and 72 °C for 1 s, performed in a LightCycler 480 II (Roche Diagnostics). Primers for mouse *ApoB* (NM_009693), *glyceraldehyde-3-phosphate dehydrogenase* (*Gapdh*; NM_008084), and *hypoxanthine guanine phosphoribosyltransferase* (*Hprt*; NM_013556) mRNAs were designed by Applied Biosystems (CA).

### Western blot analysis

For western blot analysis, 2 μl serum samples were directly collected in 18 μl homogenate buffer (20 mM Tris-HCl, pH 7.4, 0.1% SDS, 0.1% Triton X-100, 0.01% sodium deoxycholate, and 1 × complete protease inhibitor cocktail [Roche Diagnostics]). Total proteins were separated by 5% polyacrylamide gel electrophoresis and transferred onto polyvinylidenedifluoride membranes. Blots were probed with goat antibodies against apolipoprotein B (1:500; Santa Cruz Biotechnology, CA) and then incubated with horseradish peroxidase-conjugated anti-goat secondary antibodies (1:2000; Santa Cruz Biotechnology). Blots were visualized with SuperSignal West Femto Maximum Sensitivity Substrate (Thermo Fisher Scientific, MA) and analyzed with the ChemiDoc System (Bio-Rad, Hercules, CA).

### 5′-Rapid amplification of cDNA ends (RACE) analysis

Liver total RNA (5 μg) from mouse was ligated to a 5′-RACE adapter, using FirstChoice RLM-RACE Kit (Invitrogen) without prior treatment. Ligated RNA was reverse transcribed using random decamers. To detect cleavage products, the first nested PCR was performed using 5′-RACE outer primers complementary to the RNA adaptor (forward, 5′-GCTGATGGCGATGAATGAAC ACTG-3′) and *ApoB* mRNA (reverse, 5′-CTCCTGTTGCAGTAGAGTGCAGCT-3′), and the second nested PCR was performed using 5′-RACE inner primers complementary to the RNA adaptor (forward, 5′-CGCGGATCCGAACACTGCGTTTGCTGGCTTTGATG-3′) and *ApoB* mRNA (reverse, 5′-ACGCGTC GACGTGGGAGCATGGAGGTTGGCAGTTGTTC-3′). Amplification products were resolved by agarose gel electrophoresis and visualized by ethidium bromide staining.

### Northern blot analysis

For northern blot analysis, small RNA (<200 nucleotides) was extracted from mouse liver or lymph using MirVana. Small RNA was condensed with ethachinmate (Nippon Gene), and 2 μg RNA was separated by electrophoresis on 14% polyacrylamide-urea gels and transferred to Hybond-N + membranes (Amersham Biosciences, Uppsala, Sweden). Blots were hybridized with a fluorescein-labeled probe (Gene Images 3′-Oligolabelling Kit, Amersham Biosciences) for the siRNA antisense sequence or mouse U6 sequence. Signals were visualized using a Gene Images CDP-Star Detection Kit (Amersham Biosciences).

### Fast protein liquid chromatography (FPLC)

Lipoprotein profiles of mouse lymph were analyzed by an online dual enzymatic method for simultaneous quantification by FPLC at Skylight Biotech (Akita, Japan). Intestinal lymph from mouse (10 μl) was diluted with saline up to a volume of 200 μl and loaded onto FPLC columns, followed by simultaneous and continuous detection of total cholesterol and triglycerides. The flow-through was subfractionated into 87 tubes for 15 s each, with 262.5 μl in each tube. Samples were taken from each subfraction: tube 7 contained the chylomicron-sized fraction from intestinal lymph; tube 21 contained the very-low-density lipoprotein (VLDL)-sized fraction; tube 32 contained the LDL-sized fraction; and tube 46 contained the HDL-sized fraction. Each sample was used for FCS analysis.

### Statistical analysis

Experiments were replicated at least thrice. The results are presented as means ± standard error of the means (s.e.m.). The differences between means for two groups were statistically analyzed using Student’s *t*-test.

## Additional Information

**How to cite this article**: Murakami, M. *et al.* Enteral siRNA delivery technique for therapeutic gene silencing in the liver via the lymphatic route. *Sci. Rep.* **5**, 17035; doi: 10.1038/srep17035 (2015).

## Supplementary Material

Supplementary Information

## Figures and Tables

**Table 1:** Values for immune and serum biochemistry markers.

	IFN-α (pg/ml)	Cre (mg/dl)	ALT (U/L)	Na (mEq/L)	K (mEq/L)
PBS alone	<12.5	0.12 ± 0.01	26.0 ± 3.21	154 ± 1.2	4.93 ± 0.12
Toc-siRNA/MM	<12.5	0.13 ± 0.03	15.3 ± 1.76	150 ± 2.3	4.47 ± 0.58

Values represent means ± s.e.m. (*n* = 3). MM, mixed micelles; IFN-α, interferon alpha; Cre, creatinine; ALT, alanine transaminase; Na, sodium; K, potassium.
