# Supplementary material for: Enteral siRNA delivery technique for therapeutic gene silencing in the liver via the lymphatic route
Source: Sci Rep. 2015 Nov 23;5:17035. doi: 10.1038/srep17035 (PMC4655470; doi:10.1038/srep17035)
Supplement: Supplementary Information [file srep17035-s1.pdf]

Supplementary Information

Enteral siRNA delivery technique for therapeutic gene silencing in the liver via the lymphatic route

Masahiro Murakami[1,2], Kazutaka Nishina[2,3], Chie Watanabe[1,2], Kie Yoshida-Tanaka[2,3], Wenying Piao[2,3], Hiroya Kuwahara[2,3], Yuji Horikiri[1], Kanjiro Miyata[4], Nobuhiro Nishiyama[4,5], Kazunori Kataoka[4,6], Masayuki Yoshida[7], Hidehiro Mizusawa[3,8,9] & Takanori Yokota[2,3,8]

[1] Laboratory of Pharmaceutics, Faculty of Pharmacy, Osaka Ohtani University, 3-11-1 Nisikiori-Kita, Tondabayashi-shi, Osaka 584-8540, Japan.

[2] Section of Molecular Technology, Core Research for Evolutional Science and Technology (CREST), Japan Science and Technology Agency (JST), 4-1-8 Honcho, Kawaguchi-shi, Saitama 332-0012, Japan.

[3] Department of Neurology and Neurological Science, Graduate School of Medical and Dental Sciences, Tokyo Medical and Dental University, 1-5-45 Yushima, Bunkyo-ku, Tokyo 113-8519, Japan.

[4] Division of Clinical Biotechnology, Center for Disease Biology and Integrative Medicine, Graduate School of Medicine, The University of Tokyo, 7-3-1 Hongo, Bunkyo-ku, Tokyo 113-8656, Japan.

[5] Polymer Chemistry Division, Chemical Resources Laboratory, Tokyo Institute of Technology, 4259 Nagatsuta, Midori-ku, Yokohama-shi, Kanagawa 226-8503, Japan.

[6] Department of Materials Engineering, Graduate School of Engineering, The University of Tokyo, Bunkyo-ku, Tokyo 113-8656, Japan.

[7] Department of Life Science and Medical Ethics, Graduate School of Medical and Dental Sciences, Tokyo Medical and Dental University, 1-5-45 Yushima, Bunkyo-ku, Tokyo 113-8519, Japan.

[8] Center for Brain Integration Research, Tokyo Medical and Dental University, 1-5-45 Yushima, Bunkyo-ku, Tokyo 113-8519, Japan.

[9] National Center Hospital, National Center of Neurology and Psychiatry, 4-1-1 Ogawahigashi-machi, Kodaira-shi, Tokyo 187-8551, Japan.

SUPPLEMENTARY FIGURES

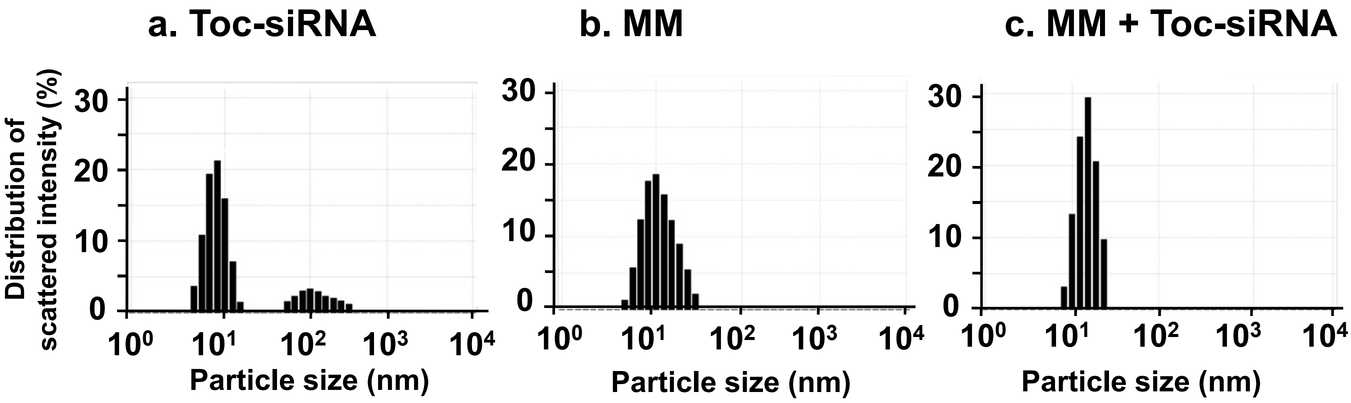

Supplementary Figure S1. Particle-size distribution was determined by dynamic light scattering (DLS) on (a) Toc-siRNA aqueous solution (pH 7.4 in PBS) and (b) linoleic acid-HCO60 mixed micelles (MM). To examine formation of complexes, Toc-siRNA was incubated in advance at 37 ° C for 30 min with MM (c). Each experiment was performed in triplicate and the representative data of distribution were shown.

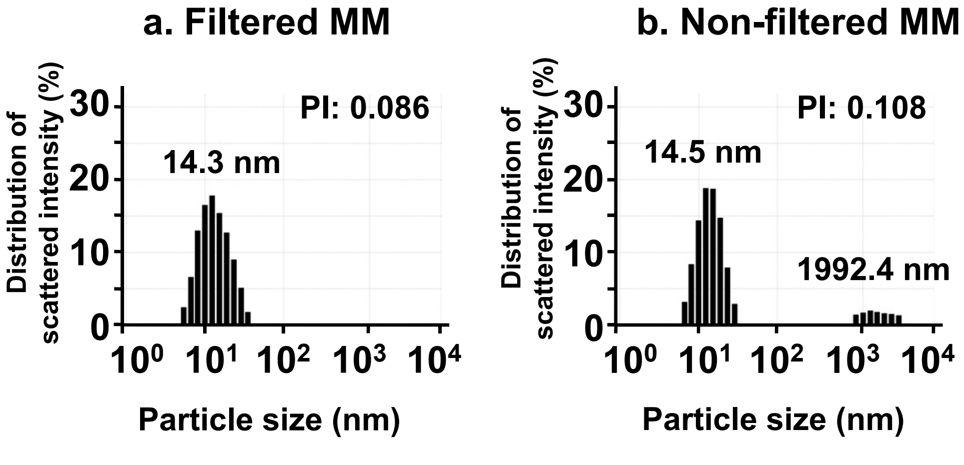

Supplementary Figure S2. Particle-size distribution of filtered mixed micelles (a) and unfiltered mixed micelles (b).

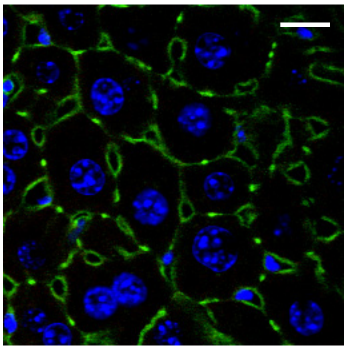

Supplementary Figure S3. Confocal images of mouse livers 4 h after administration of LNP to the jejunum. The dose of Toc-siRNA was 10 mg/kg of body weight. Red: Cy3-labeled Toc-siRNA; Green: FITC-Phalloidin; Blue: TO-PRO-3. Bar = 20 μm.

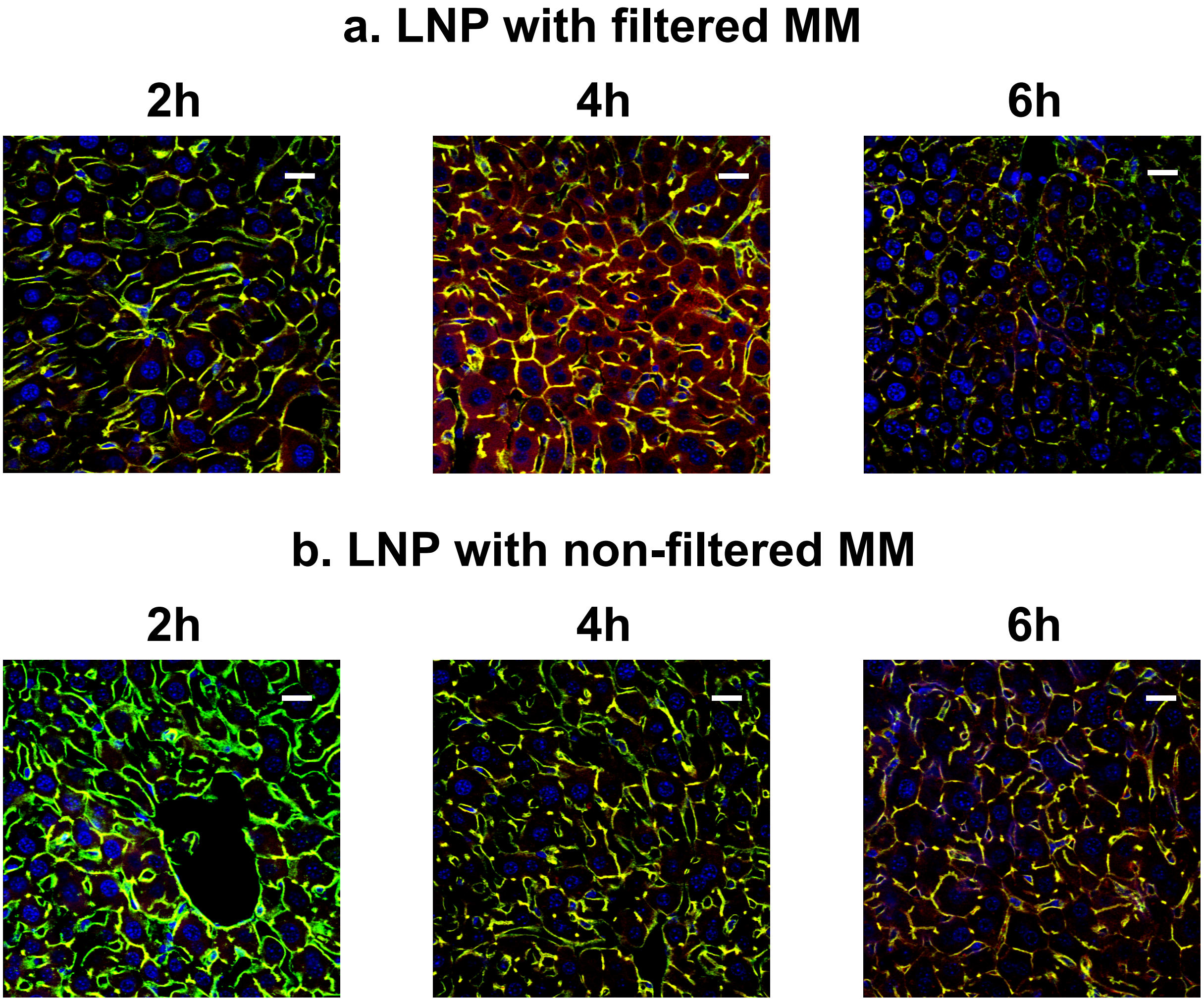

Supplementary Figure S4. Size distribution effect of emulsification on Toc-siRNA delivery. Confocal images of mouse liver at 2, 4, and 6 h after colorectal administration of LNP constructed with filtered mixed micelles (a) or with unfiltered mixed micelles (b). The uniformly-sized nano-emulsification was likely essential for effective siRNA delivery. Red, Cy3-labeled Toc-siRNA; Green: FITC-Phalloidin; Blue, TO-PRO-3. Bar = 20 μm.

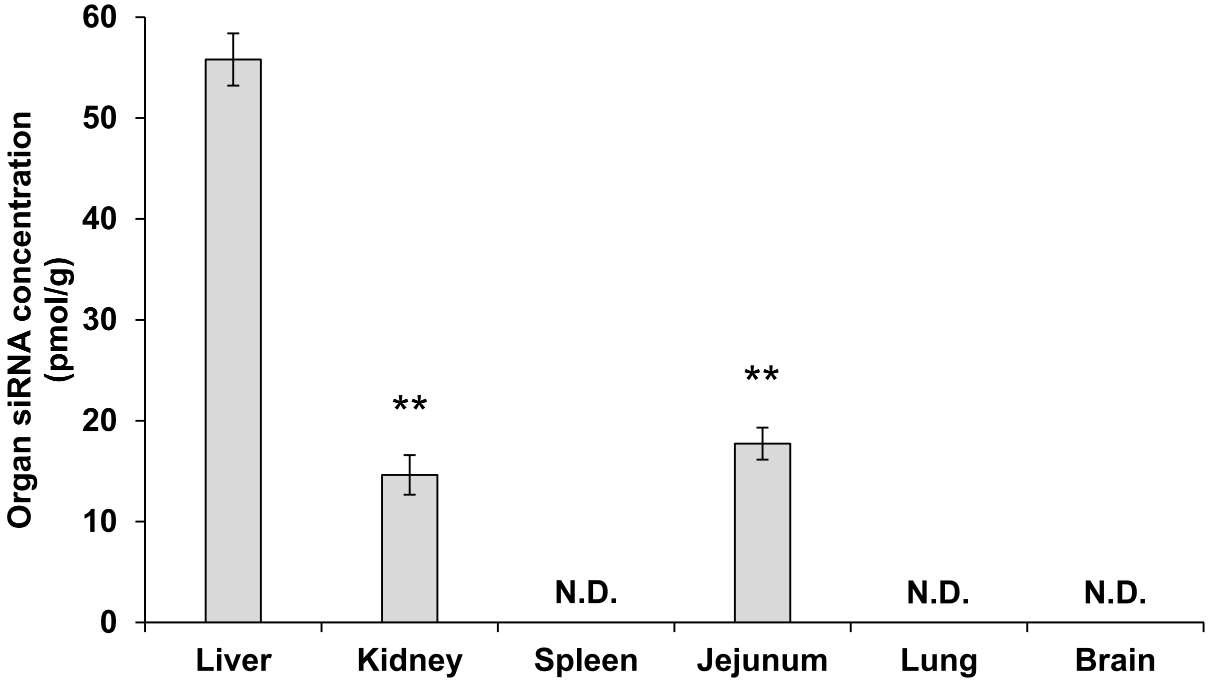

Supplementary Figure S5. Concentration of Toc-siRNA in each organ 4 h after administration of 10 mg/kg of Cy3–labeled Toc-siRNA. *n* = 3, mean values ± s.e.m; ** *P* < 0.01 versus Liver. N.D.: not detected.

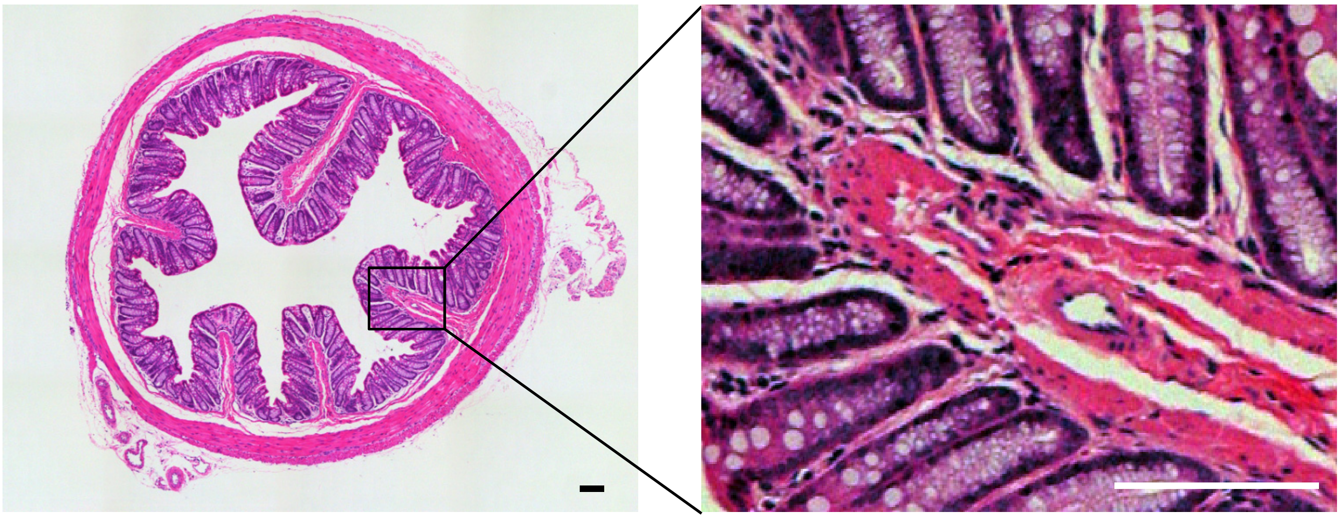

Supplementary Figure S6. Histological analysis by hematoxylin-eosin staining of the colon of the mouse after colorectal administration of Toc-siRNA with linoleic-acid mixed micelle. No pronounced local damage or histologic abnormality was observed. Bar = 100 µm.

SUPPLEMENTARY TABLES

Supplementary Table S1. Mean diameter and size distribution of particles determined by DLS.

Composition	Mean diameter (nm)		The peak average (nm) Scattered light distribution		Number distribution		Volume distribution		Polydispersity index	
Linoleic acid-HCO60 mixed micelle	14.2 ± 0.2	**	17.1 ± 2.2	NS	8.7 ± 1.5	NS	8.6 ± 1.6	NS	0.133 ± 0.011	NS
Mixed micelle + Toc-siRNA	15.1 ± 0.1		15.6 ± 0.4		9.1 ± 0.7		11.4 ± 0.4		0.103 ± 0.021	
Lymph	267.8 ± 4.3	**	366.4 ± 8.3	*	141.2 ± 18.1	NS	185.7 ± 22.9	NS	0.235 ± 0.008	**
Lymph + Toc-siRNA	238.5 ± 1.6		287.7 ± 17.1		127.8 ± 4.8		167.9 ± 6.9		0.185 ± 0.014	
Chylomicron fraction (CM)	148.2 ± 2.0	**	172.7 ± 2.0	NS	88.7 ± 8.6	NS	112.7 ± 7.8	NS	0.133 ± 0.019	NS
CM + Toc-siRNA	158.3 ± 1.2		179.9 ± 5.3		100.6 ± 3.5		125.3 ± 6.7		0.133 ± 0.007	

Mixed micelles comprised linoleic acid and HCO60 were filtered through a membrane filter (pore size, 0.45 μm). Lymph was collected via a thoracic duct fistula of the mice under the postprandial conditions, followed by ultracentrifugation to obtain the chylomicron fraction (CM). Toc-siRNA was incubated *in vitro* with MM, lymph, and CM at 37°C for 30 min. The particle size and size distribution were determined by DLS (Photal ELSZ-2). All samples in this table showed only one peak in the size distribution. The peak diameter and polydispersity index were calculated by a cumulant method. The data were presented as the means ± s.e.m. for independently triplicate determinations. Statistical analysis was performed by Student's *t*-test; * $p < 0.05$; ** $p < 0.01$; NS, not significant.

Supplementary Table S2. Concentration of Toc-siRNA in mouse liver and serum after rectal or intravenous administration of Toc-siRNA.

Organ	Administraion route	Liver (pmol/g) / Serum (nmol/l)	
Liver	Intrarectal	55.8 ± 2.6	**
	Intravenous	528.1 ± 138.0	
Serum	Intrarectal	8.20 ± 0.65	**
	Intravenous	647.08 ± 100.25	

The data were presented as the means ± s.e.m. for independently triplicate determinations. Statistical analysis was performed by Student's *t*-test; ** $p < 0.01$.

Supplementary Table S3. Sequences of Toc-siRNA.

Toc-siRNA	Strand	Sequence
Toc-siApoB	Sense	5′-GuCAuCACACuGAAuACCAAUGCugG*A-3′
(targeting mouse *ApoB* mRNA: NM_009693)	Antisense	5′-ucc*A*gc*AUUGGuAuUCAGUGuGAuGAc*A*C-3′
Toc-siOAT3	Sense	5′-CcAUuAUCUUgAAUgUGGAAUGGguA*c-3′
(targeting mouse *Oat3* mRNA: NM_031194)	Antisense	5′-gua*C*gc*AUUCCaCaUUCAAGaUAaUGg*U*G-3′
Toc-siScr	Sense	5′-AcAUuUUACCuGGCaAGGAGUCAauC*C-3′
(Scrambled sequence of Toc-siApoB)	Antisense	5′-gga*U*ug*ACUCCuUgCCAGGUaAUaUGu*A*C-3′

Upper case letters represent RNA, lower case letters represent 2′-*O*-methyl sugar modification, and asterisks represent phosphorothioate linkages.

SUPPLEMENTARY METHODS

Particle sizing by dynamic light scattering (DLS). Particle size distribution was evaluated by DLS (PhotalELSZ-2, Otsuka Electronics Co. Ltd., Osaka, Japan). Toc-siRNA was incubated *in vitro* with linoleic acid-HCO60 mixed micelles at 37°C for 30 min, and formation of complexes was confirmed by DLS.

Concentration of Toc-siRNA in each organ. In rectal administration, mice were administered with Cy3–conjugated Toc-siRNA, and 4 h later various organs (liver, kidney, spleen, jejunum, lung and brain) and serum were harvested. In intravenous injection, mice were injected from tail vein with Cy3–conjugated Toc-siRNA, and 1 h later liver and serum were harvested. The signal intensity of Cy3 in the samples was measured by i-control (Tecan, Männedorf, Switzerland), then the concentration of Toc-siRNA was calculated.
